# Pathogenesis-Informed Phenotype-Guided Therapy for MASH: A Three-Axis Translational Framework Within the MASLD Spectrum

**DOI:** 10.3390/biomedicines14091884

**Published:** 2026-08-24

**Authors:** Zishu Zhao, Xiaoyang Hu

**Affiliations:** 1 Graduate School, Heilongjiang University of Chinese Medicine, Harbin 150040, China; wansui2619@163.com; 2 Basic Medical College, Heilongjiang University of Chinese Medicine, Harbin 150040, China

**Keywords:** non-alcoholic fatty liver disease (MeSH), metabolic dysfunction-associated steatohepatitis (MeSH entry term), drug therapy (MeSH), fibrosis (MeSH), translational research, biomedical (MeSH), phenotype-guided therapy (free-text term), metabolic unloading (free-text term), lipid reprogramming (free-text term)

## Abstract

Metabolic dysfunction-associated steatohepatitis (MASH) is the progressive inflammatory and fibrotic subtype of metabolic dysfunction-associated steatotic liver disease (MASLD), and its treatment landscape is rapidly moving from nonspecific liver fat reduction towards mechanism-based drug positioning. Since 2024, resmetirom and semaglutide have received U.S. Food and Drug Administration accelerated approval for non-cirrhotic MASH with moderate-to-advanced fibrosis, while tirzepatide, survodutide, and fibroblast growth factor 21 analogues have shown biopsy-based phase 2 or 2b efficacy signals. This narrative review organises approved and emerging pharmacotherapies within a pathogenesis-informed three-axis framework: the weight-insulin resistance-substrate load axis, the intrahepatic lipid reprogramming axis, and the inflammation-fibrosis transition and multi-axis integration axis. We further grade evidence maturity from regulatory or phase 3 histological evidence to phase 2 biopsy-based evidence and earlier imaging- or biomarker-based signals. This framework is intended to support phenotype-sensitive treatment positioning rather than a fixed therapeutic sequence. In particular, weight-centred treatment should not be assumed to apply to all patients, including normal-weight or lean MASH. Overall, future MASH therapy will likely depend on matching drug mechanisms, fibrosis stage, cardiometabolic phenotype, and treatment goals.

## 1. Introduction

Metabolic dysfunction-associated steatotic liver disease (MASLD) is a chronic metabolic liver disease characterised by the coexistence of hepatic steatosis and at least one cardiometabolic risk factor [1]. Within this spectrum, metabolic dysfunction-associated steatohepatitis (MASH) is an active lesion characterised by the presence of hepatocellular ballooning and lobular inflammation on a background of hepatocellular steatosis, with or without varying degrees of fibrosis, and represents a key subtype at higher risk of fibrosis progression in the MASLD spectrum [2,3].

Epidemiological studies published in the last decade have shown that the disease burden and incidence of steatotic liver disease are continuously increasing, making it an important issue in global chronic liver disease prevention and control [4,5,6]. In terms of disease evolution, a subset of patients with MASLD may progress to MASH, and the persistent inflammatory injury accompanying MASH can further promote the occurrence and progression of liver fibrosis [7,8]. Given the above pathological processes, the therapeutic goals for MASLD/MASH should not be limited to reductions in transaminases or imaging-based fat reduction, but rather, on the basis of lowering hepatic fat burden, further attention should be paid to the alleviation of lipotoxicity, inhibition of inflammation, MASH resolution, regression or stabilisation of fibrosis, and simultaneous improvement in body weight, blood glucose, blood lipids, and related cardiometabolic risks [9,10].

For a long time, drug interventions for the MASLD spectrum have predominantly used improvements in liver fat content, transaminase levels, and other imaging or biochemical markers as primary endpoints, and treatment options that have truly achieved key histological endpoints and entered the regulatory approval stage remain limited. Since the 2024 FDA accelerated approval of resmetirom, substantial progress has been made in drug research for MASH, particularly in patients with non-cirrhotic F2–F3 fibrosis. Among these, resmetirom received FDA accelerated approval in 2024 for the treatment of adults with non-cirrhotic MASH with moderate-to-advanced liver fibrosis, with its core histological evidence derived from the phase 3 MAESTRO-NASH trial (that study used the previous nomenclature of NASH, which corresponds to MASH under the current classification system) [11,12]; semaglutide 2.4 mg also subsequently received FDA accelerated approval based on positive histological results from the phase 3 ESSENCE study [13,14]. At the same time, tirzepatide, survodutide, and FGF21 analogues have shown positive histological signals in phase 2 or 2b studies [15,16,17,18], and relevant subsequent phase 3 studies or clinical outcome trials are also underway. These advances indicate that the therapeutic evidence for MASH within the MASLD spectrum is shifting from simple biochemical or imaging improvements towards a registration research paradigm centred on histological endpoints. Correspondingly, clinical treatment discussions are no longer limited to lifestyle intervention and liver fat reduction, but are beginning to pay more attention to drug evidence strength, histological endpoints, and therapeutic positioning across different pathological stages and metabolic phenotypes.

The reviews and position statements cited here, published between 2019 and 2024, have discussed MASLD/MASH pharmacotherapies from drug-class, liver-targeted metabolic, cardiometabolic, and personalised-treatment perspectives [19,20,21,22]. In particular, Rinella and Sookoian emphasised in their 2026 review the need to integrate therapeutic targets, fibrosis stage, metabolic phenotype, and cardiometabolic burden when considering personalised disease management in MASH [22]. The present review therefore does not aim to claim that phenotype-based treatment positioning is a new concept. Instead, it provides a complementary translational framework that organises available and emerging agents along three pathobiological axes: weight-insulin resistance-substrate load, intrahepatic lipid reprogramming, and inflammation-fibrosis transition with multi-axis integration. In addition, the A–C evidence-maturity grading used here is intended to help readers distinguish regulatory or phase 3 histological evidence from phase 2 biopsy-based evidence and earlier imaging- or biomarker-based signals.

## 2. Literature Selection and Evidence Interpretation

This is a narrative review. We prioritised regulatory documents, phase 2/2b and phase 3 randomised controlled trials with biopsy-based or imaging-based endpoints, major clinical guidelines, systematic reviews, and meta-analyses published mainly from 2024 to 2025, and landmark mechanistic studies relevant to MASLD/MASH pathogenesis and pharmacotherapy. Drugs were discussed according to their dominant pharmacological mechanism, clinical translation stage and available evidence for histological or metabolic endpoints. The A–C evidence maturity levels used in this review are intended for comparative narrative interpretation and should not be regarded as formal GRADE recommendations. The contemporaneous review by Rinella and Sookoian was specifically considered when defining the scope and novelty boundary of this narrative synthesis, and the present framework was positioned as complementary rather than competing with that personalised-treatment model [22].

## 3. Pathological Basis of Metabolism-Directed Therapy for MASH in the MASLD Spectrum and the Three-Axis Framework

**Figure 1:**
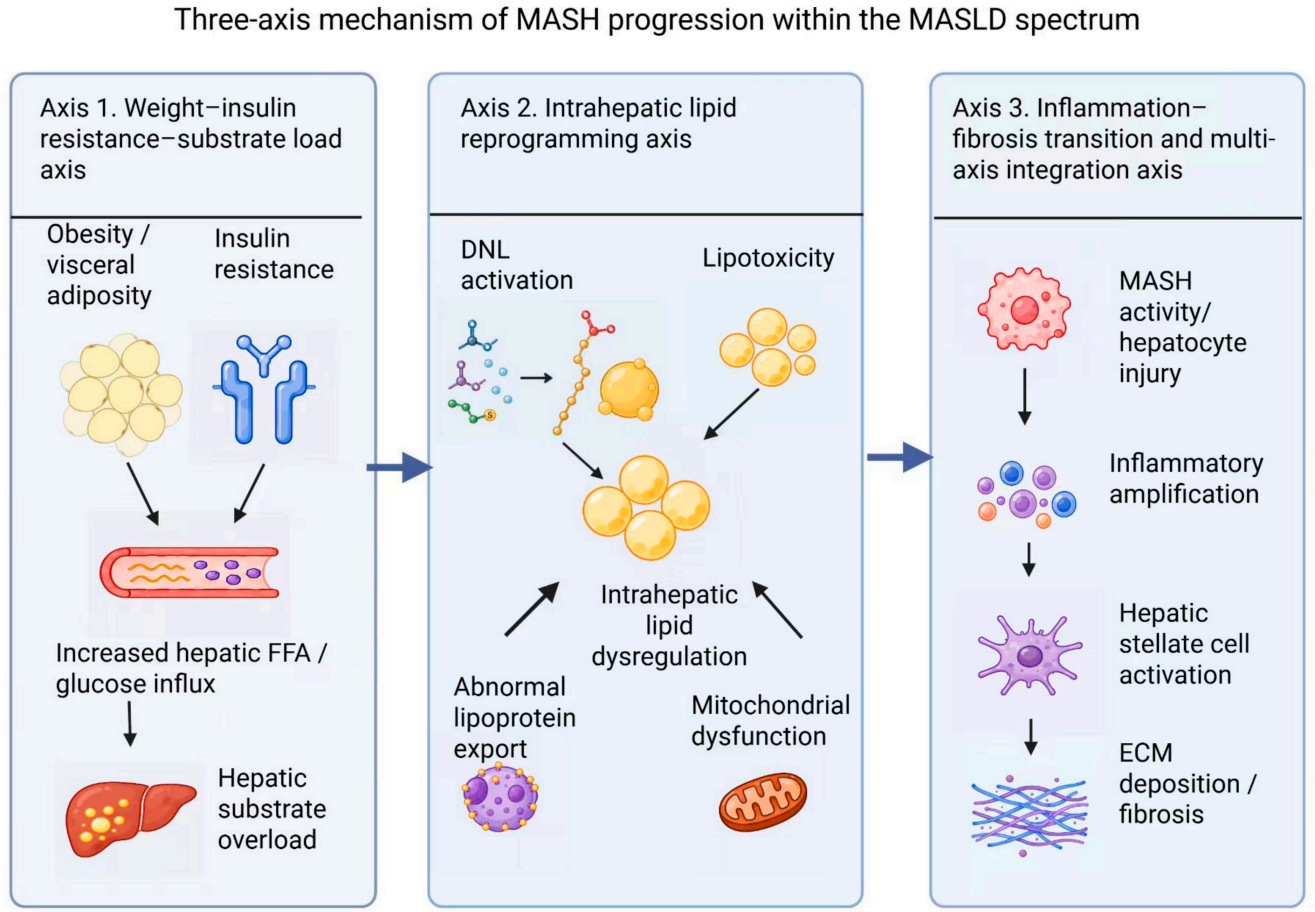
Pathogenesis-informed three-axis framework for phenotype-guided therapy of MASH within the MASLD spectrum. The framework summarises three interrelated therapeutic axes: the weight–insulin resistance–substrate load axis, the intrahepatic lipid reprogramming axis, and the inflammation–fibrosis transition and multi-axis integration axis. These axes are intended to indicate dominant therapeutic positioning rather than mutually exclusive mechanisms, because many agents may act across more than one pathological pathway. DNL, de novo lipogenesis; ECM, extracellular matrix; FFA, free fatty acid; MASH, metabolic dysfunction-associated steatohepatitis; MASLD, metabolic dysfunction-associated steatotic liver disease.

Progression from MASLD to MASH and fibrosis is rarely due to a single abnormality. Instead, it is closely linked to systemic metabolic dysregulation, intrahepatic lipid handling imbalance, inflammatory immune activation, and persistent amplification of fibrotic responses [23,24]. Obesity, visceral fat accumulation and insulin resistance can increase the input of free fatty acids and other metabolic substrates to the liver, generating upstream metabolic pressure. Enhanced de novo lipogenesis within hepatocytes, imbalance in fatty acid oxidation, and accumulation of lipotoxic intermediates can further disturb intrahepatic lipid synthesis, oxidation, storage and export processes, and induce organellar stress. When lipotoxicity, oxidative stress, and innate immune responses persist, the disease can progress from simple steatosis to active MASH lesions, accompanied by hepatic stellate cell activation and fibrosis progression. Accordingly, this review summarises the pathological basis of metabolism-directed therapy for MASH within the MASLD spectrum into three analytical dimensions: the weight–insulin resistance–substrate load axis, the intrahepatic lipid reprogramming axis, and the inflammation-fibrosis transition and multi-axis integration axis. The three-axis framework is therefore intended as a dominant-positioning framework rather than as an exclusive drug-classification system. Its discriminative value lies in identifying the main pathological bottleneck and therapeutic rationale emphasised by each drug or drug class: upstream metabolic unloading, intrahepatic lipid reprogramming, or the transition from inflammatory activity to fibrosis progression. Cross-axis effects are expected in MASH pharmacotherapy and do not invalidate the framework; instead, they help indicate where a drug may be best positioned as monotherapy, combination therapy, or sequential therapy according to patient phenotype and evidence maturity. Importantly, the weight–insulin resistance–substrate load axis should not be interpreted as implying that weight loss is the therapeutic foundation for all patients with MASH, particularly in normal-weight or “lean” MASLD/MASH, in whom treatment selection should be individualised according to metabolic phenotype, visceral adiposity, fibrosis risk, and alternative disease-driving mechanisms [25,26,27]. Based on regulatory approval status, phase 3 histological evidence, phase 2/2b histological evidence, and imaging or exploratory endpoints, relevant drugs are assigned A–C evidence maturity levels. This stratification is used only for comparative review purposes in this article and is not equivalent to a formal GRADE assessment or guideline recommendation level. The three-axis pathological mechanism framework is shown in Figure 1, and representative drugs, relevant clinical phenotypes, and evidence maturity stratification are shown in Table 1. For clinical interpretation, the assignment of a patient phenotype to a dominant axis should be based on commonly used clinical and non-invasive indicators rather than on a single variable. The weight–insulin resistance–substrate load axis is most relevant when obesity or overweight, increased waist circumference or visceral adiposity, type 2 diabetes, prediabetes, metabolic syndrome, elevated fasting glucose, or insulin resistance is present. The intrahepatic lipid reprogramming axis is more relevant when dyslipidaemia, elevated triglycerides or LDL-C, high hepatic fat burden on imaging, or evidence of altered hepatic lipid handling is prominent. The inflammation–fibrosis transition and multi-axis integration axis is more relevant when there is biopsy-confirmed MASH activity, F2–F3 fibrosis, high-risk non-invasive fibrosis assessment, elevated liver stiffness, or clinical concern for fibrosis progression [2,26,28]. These criteria are intended to guide phenotype-based therapeutic positioning and should not be interpreted as strict diagnostic cut-offs or treatment eligibility criteria.

### 3.1. Weight–Insulin Resistance–Substrate Load Axis

The first axis primarily reflects the upstream metabolic drivers in the progression from MASLD to MASH. Excessive energy intake, visceral adiposity, and insulin resistance increase the delivery of metabolic substrates such as free fatty acids and glucose to the liver, accompanied by hyperinsulinaemia and abnormal glucose and lipid fluxes, ultimately leading to hepatic substrate overload [10,49]. The focus of such interventions is not primarily to directly reverse established fibrosis, but rather to reduce subsequent lipotoxicity and inflammatory burden by lowering body weight, improving insulin sensitivity, and decreasing hepatic substrate input. Among these, glucagon-like peptide-1 (GLP-1) receptor agonists, glucose-dependent insulinotropic polypeptide/glucagon-like peptide-1 (GIP/GLP-1) dual receptor agonists, sodium-glucose cotransporter 2 (SGLT2) inhibitors, and pioglitazone can be mainly classified under this metabolic unloading pathway [50].

### 3.2. Intrahepatic Lipid Reprogramming Axis

The second axis emphasises the imbalance in hepatic lipid synthesis, oxidation, storage, and export processes. Enhanced de novo lipogenesis (DNL), abnormal lipid droplet remodelling, accumulation of lipotoxic intermediates, and mitochondrial stress together exacerbate hepatocellular lipotoxicity and organelle stress [51]. Drugs targeting this axis are more closely related to intrahepatic lipogenesis, lipid clearance, fatty acid oxidation and lipotoxicity alleviation, such as thyroid hormone receptor-β (THR-β) agonists, fibroblast growth factor 21 (FGF21) analogues, fatty acid synthase (FASN), acetyl-CoA carboxylase (ACC), and diacylglycerol O-acyltransferase 2 (DGAT2) inhibitors [20,52].

### 3.3. Inflammation–Fibrosis Transition and Multi-Axis Integration Axis

The third axis corresponds to the pathological process by which metabolic stress transitions to MASH activity and fibrosis progression within the MASLD spectrum. When upstream metabolic disturbances and intrahepatic lipid handling imbalance persist, ongoing lipotoxicity further induces oxidative stress and innate immune activation, promoting Kupffer cell activation, inflammatory cytokine release, and hepatic stellate cell activation, thereby facilitating extracellular matrix deposition and fibrosis progression [53,54]. At this stage, simply reducing upstream metabolic load may be insufficient, and therapeutic strategies with broader multi-axis coverage may be required. Survodutide and certain FGF21 analogues have shown relatively positive signals in MASH histological endpoints or fibrosis improvement, representing important development directions for multi-axis integrative therapy; multi-receptor agonists such as retatrutide have mainly demonstrated potential for liver fat reduction and improvement of related metabolic markers, but their effects on MASH histological endpoints and fibrosis improvement require further validation in subsequent studies. Overall, the independent contribution of the anti-fibrotic effects of these drugs, separate from upstream metabolic improvements such as weight loss and liver fat reduction [29], remains incompletely defined and needs to be distinguished by future mechanistic studies and long-term clinical trials [15,16]. Accordingly, the three-axis framework should not be interpreted as a rigid drug-classification system, because many agents act across more than one biological pathway. Its purpose is to provide a pragmatic interpretive structure for linking dominant pathological drivers, candidate patient phenotypes, and evidence maturity, while acknowledging that multi-axis effects are common and clinically important.

## 4. Drugs Targeting the First Axis: Promoting Weight Loss, Improving Insulin Sensitivity, and Reducing Hepatic Substrate Load

### 4.1. GLP-1 Receptor Agonists

GLP-1 receptor agonists primarily act by suppressing appetite, delaying gastric emptying, reducing body weight, and improving insulin sensitivity, thereby decreasing the delivery of free fatty acids and other metabolic substrates from adipose tissue to the liver, which in turn reduces intrahepatic lipid deposition and subsequent inflammatory burden [55]. A phase 2 study of semaglutide showed that semaglutide significantly increased the proportion of patients achieving MASH resolution without worsening of fibrosis, but the advantage in fibrosis improvement did not reach statistical significance [56]. Subsequently, the phase 3 ESSENCE trial further strengthened the histological evidence: after 72 weeks of treatment with once-weekly semaglutide 2.4 mg, the proportion of patients achieving MASH resolution without worsening of fibrosis was 62.9%, compared with 34.3% in the placebo group; the proportion achieving ≥1-stage fibrosis improvement without worsening of MASH was 36.8%, compared with 22.4% in the placebo group [13]. Based on the planned interim analysis results of the phase 3 ESSENCE trial, semaglutide 2.4 mg received FDA accelerated approval in 2025 for adults with non-cirrhotic MASH with moderate-to-advanced fibrosis [13,14]; however, this approval is still based on histological surrogate endpoints (MASH resolution and fibrosis improvement), and further confirmatory studies are needed to validate its effects on clinical outcomes such as cirrhosis progression, liver-related events, liver transplantation, and mortality [57].

Beyond histological endpoints, real-world studies have provided complementary evidence for the long-term benefits of GLP-1 receptor agonists. A US Veterans cohort study showed that, among patients with MASLD and diabetes who had not yet progressed to cirrhosis, GLP-1 receptor agonist use was associated with a lower risk of cirrhosis progression and death [58]. Another multinational real-world study also suggested that, in patients with MASLD and type 2 diabetes, GLP-1 receptor agonists were associated with a lower risk of major adverse liver outcomes and all-cause mortality compared with SGLT2 inhibitors [59]. However, the above evidence is mainly derived from observational studies, and causality as well as long-term clinical benefits still require further validation.

### 4.2. GIP/GLP-1 Dual Receptor Agonists

Tirzepatide is a representative drug of the GIP/GLP-1 dual receptor agonist class. Compared with selective GLP-1 receptor agonists, its simultaneous action on the GIP and GLP-1 pathways confers distinct metabolic intervention characteristics in promoting weight loss, improving insulin sensitivity, and regulating energy metabolism, thus offering considerable therapeutic and developmental potential in obesity- or insulin resistance-dominant MASH phenotypes [60,61,62]. The phase 2 SYNERGY-NASH study showed that after 52 weeks of treatment, the proportions of patients achieving MASH resolution without worsening of fibrosis in the 5 mg, 10 mg, and 15 mg tirzepatide groups were 44%, 56% and 62%, respectively, all higher than the 10% in the placebo group; the proportions achieving ≥1-stage fibrosis improvement without worsening of MASH were 55%, 51%, and 51%, respectively, also higher than the 30% in the placebo group [18]. Further post hoc subgroup analyses indicated that the direction of histological improvement with tirzepatide was generally consistent across different clinical subgroups, suggesting that its treatment effects are not confined to a single patient profile; however, these results remain exploratory and require larger sample sizes and longer-term studies for validation [30]. These findings suggest that the action of tirzepatide is not limited to weight loss, and its improvement of MASH histological endpoints has received preliminary support from phase 2 studies. Within the three-axis framework, although tirzepatide is mainly classified under the first axis, its marked weight-lowering and insulin-sensitising effects may also further influence intrahepatic lipid reprogramming and the inflammation–fibrosis transition process.

### 4.3. SGLT2 Inhibitors

SGLT2 inhibitors have previously been discussed mainly as agents for managing metabolic comorbidities in patients with type 2 diabetes and MASLD, and evidence for their effects on MASH histological endpoints has been relatively limited. A randomised controlled trial published in 2025 in The BMJ, which used liver biopsy to assess histological endpoints, further supplemented clinical evidence in this area. This study showed that after 48 weeks of dapagliflozin treatment, the proportion of patients achieving MASH improvement without worsening of fibrosis was 53%, compared with 30% in the placebo group; the proportion achieving MASH resolution was 23% vs. 8%; and the proportion achieving fibrosis improvement was 45% vs. 20% [31]. Meanwhile, a 2025 meta-analysis suggests that SGLT2 inhibitors reduce liver fat content and may decrease hepatic metabolic burden by improving body weight, glycaemic control, and fat distribution [32]. Therefore, in type 2 diabetes-dominant MASLD phenotypes, SGLT2 inhibitors may provide not only glucose-lowering and cardiorenal benefits, but also complementary benefits in reducing hepatic metabolic burden.

### 4.4. Other Insulin-Sensitising Strategies

Among other insulin-sensitising strategies, the peroxisome proliferator-activated receptor-γ (PPAR-γ) agonist pioglitazone, although not a newly developed drug, retains an evidence base in MASH patients with marked insulin resistance, prediabetes, or type 2 diabetes. Previous randomised controlled studies have shown that pioglitazone improves NASH histological activity and promotes improvements in inflammation and steatosis in a subset of patients [33,34]. A 2025 multicentre preliminary study also suggests that long-term pioglitazone treatment may be associated with improvements in non-invasive parameters such as liver enzymes, hepatic steatosis, and FAST score, but these findings require validation in higher-quality studies [35]. However, its clinical use remains limited by concerns over weight gain, oedema, fracture risk, and long-term safety and tolerability. Therefore, pioglitazone is better positioned as a stratified alternative for patients with specific metabolic phenotypes.

## 5. Drugs Targeting the Second Axis: Modulating Intrahepatic Lipid Metabolism and Lipotoxicity

### 5.1. THR-β Agonist Resmetirom

Resmetirom is currently the most clinically advanced agent within the second axis and represents a liver-targeted metabolic therapy with a relatively advanced level of clinical translation. As a selective hepatic THR-β agonist, resmetirom regulates intrahepatic lipid metabolism, reduces hepatic fat accumulation, and improves atherogenic lipid parameters such as low-density lipoprotein cholesterol (LDL-C) and triglycerides. The phase 3 MAESTRO-NASH study showed that after 52 weeks of treatment with 80 mg and 100 mg resmetirom, 25.9% and 29.9% of patients, respectively, achieved MASH resolution without worsening of fibrosis, compared with 9.7% in the placebo group; the proportions achieving ≥1-stage fibrosis improvement without worsening of MASH were 24.2% and 25.9%, respectively, also higher than the 14.2% in the placebo group [11]. Based on these histological results, the FDA granted accelerated approval in 2024 for resmetirom in adults with non-cirrhotic MASH with moderate-to-advanced fibrosis; however, this approval is still based on histological surrogate endpoints, and confirmatory studies are needed to validate its effects on clinical outcomes such as hepatic decompensation, liver transplantation, and liver-related mortality [11,12]. The American Association for the Study of Liver Diseases (AASLD) practice update and expert recommendations further indicate that clinical use of resmetirom should focus on identification of patients with F2–F3 fibrosis, lipid profile characteristics, drug interactions, and safety monitoring during treatment [36,37,63].

### 5.2. FGF21 Analogues

FGF21 analogues possess both intrahepatic lipid-regulating and cross-axis metabolic effects. In this review, based on the relatively prominent signals for liver fat reduction, metabolic improvement, and fibrosis improvement in existing evidence, they are discussed under the second axis. Unlike resmetirom, which mainly focuses on intrahepatic lipid metabolic regulation, FGF21 analogues simultaneously affect hepatic fat burden, insulin sensitivity, lipid metabolism, and inflammation–fibrosis-related pathways, representing a direction that combines “intrahepatic lipid reprogramming” with systemic metabolic improvement [47]. The phase 2b study of pegozafermin published in NEJM in 2023 showed that this drug improved fibrosis and reduced hepatic fat burden [17]. The 96-week results of the HARMONY trial reported in The Lancet in 2025 further suggested that efruxifermin showed sustained fibrosis-improvement signals over a longer treatment duration [15,39]. A 2025 systematic review and meta-analysis also supports that FGF21 analogues are superior to placebo in MASH resolution, fibrosis improvement, and magnetic resonance imaging–proton density fat fraction (MRI-PDFF)-assessed liver fat reduction, but the existing evidence for fibrosis improvement is mainly derived from non-cirrhotic fibrotic populations and still requires further confirmation in phase 3 studies [48]. At the time of manuscript preparation, both pegozafermin and efruxifermin have phase 3 programmes underway; therefore, these drugs should no longer be described simply as “early-stage exploration” based on previous phase 2b literature, but are better characterised as “having entered phase 3 development on the basis of positive phase 2b evidence” [38,40,41]. Based on current evidence, these drugs are more suitable for further validation in populations with high hepatic fat burden, pronounced triglyceride abnormalities, or a higher risk of fibrosis progression.

### 5.3. Drugs Targeting DNL and Triglyceride Synthesis

Unlike the metabolic pleiotropy of FGF21 analogues, drugs targeting DNL and triglyceride synthesis place greater emphasis on direct intervention in intrahepatic lipogenesis and lipid storage. These agents primarily reduce hepatocyte lipid accumulation and lipotoxic burden by inhibiting intrahepatic de novo lipogenesis, fatty acid synthesis, or triglyceride synthesis, representing a mechanistically specific therapeutic direction within the second axis [20]. The phase 2b study of denifanstat in 2024 suggested that inhibition of fatty acid synthase (FASN) produced statistically significant and clinically meaningful histological improvement [42]. The 2025 MIRNA study showed that the diacylglycerol O-acyltransferase 2 (DGAT2) inhibitor ervogastat, as monotherapy and in combination with the acetyl-CoA carboxylase (ACC) inhibitor clesacostat, both demonstrated signals of efficacy [43]. However, further development of such drugs still faces challenges regarding durability of efficacy, safety, and positioning in combination therapy. Therefore, drugs targeting DNL and triglyceride synthesis are better positioned at this stage as potential directions for combination therapy, and their value as monotherapy requires further confirmation in subsequent studies. Beyond DNL-related targets, the pan-PPAR agonist lanifibranor has also shown potential for improving MASH activity and fibrosis in a phase 2b study, serving as an important addition to the metabolic nuclear receptor direction [64].

## 6. Drugs Targeting the Third Axis: Inflammation–Fibrosis Transition and Multi-Axis Integration

### 6.1. GLP-1/GCGR Dual Receptor Agonist Survodutide

Survodutide is a dual receptor agonist targeting glucagon-like peptide-1 (GLP-1) and the glucagon receptor (GCGR), and is a representative multi-axis integrative agent within the third axis [16]. Its mechanism of action is not limited to body weight reduction; it may also influence MASH progression by improving energy metabolism, promoting liver fat reduction, and modulating inflammation–fibrosis-related processes.

A phase 2 study published in the NEJM showed that, after 48 weeks of treatment, MASH improvement without worsening of fibrosis was achieved in 47%, 62%, and 43% of patients in the 2.4 mg, 4.8 mg, and 6.0 mg groups, respectively, compared with 14% in the placebo group. Fibrosis improvement of at least one stage was achieved in 34%, 36%, and 34% of patients, respectively, compared with 22% in the placebo group [16]. A 2025 systematic review and meta-analysis also suggests that GLP-1-based therapies can reduce liver fat content and improve parameters such as steatosis, hepatocyte ballooning, and lobular inflammation, although evidence for fibrosis improvement remains to be further established [65].

Of note, the dose–response relationship of survodutide was not strictly linear: MASH improvement without worsening of fibrosis was achieved in 62% of patients in the 4.8 mg group, but in 43% of patients in the 6.0 mg group, suggesting that histological benefit cannot be inferred simply from the magnitude of body weight loss. At the time of manuscript preparation, the phase 3 LIVERAGE study of survodutide in MASH was ongoing; therefore, survodutide is better described as having positive phase 2 histological evidence and being under phase 3 validation [44]. Although survodutide is discussed under the third axis in this review, its effects are not confined to the inflammation–fibrosis transition step; body weight loss, liver fat reduction, and regulation of energy metabolism may collectively contribute to its therapeutic efficacy.

### 6.2. Earlier-Stage Multi-Axis Integrative Drugs

Although efinopegdutide and retatrutide mechanistically fit the multi-axis integrative characteristics of the third axis, their MASH histological evidence is not yet mature at present, and related studies are more focused on liver fat reduction, imaging endpoints, or the stage of advancing to pivotal studies. Therefore, this section discusses them separately as earlier-stage multi-axis integrative drugs. Compared with selective GLP-1 receptor agonists, these multi-receptor agonists can simultaneously modulate GLP-1-, GIP-, or GCGR-related pathways, thereby further affecting energy metabolism, adipose tissue–liver substrate flux, hepatic lipid oxidation and inflammation-related processes, and thus should be discussed under the framework of multi-axis integrative therapy [66]. A head-to-head study of efinopegdutide showed that, in patients with nonalcoholic fatty liver disease (NAFLD), its effect on reducing liver fat content after 24 weeks of treatment was superior to that of semaglutide 1 mg, but current evidence is still mainly derived from imaging endpoints [45]. Retatrutide, as a GIP/GLP-1/GCGR triple receptor agonist, demonstrated marked liver fat reduction in a 2024 study, but key histological data remain less mature than those for semaglutide, tirzepatide, or survodutide [29]. Biopsy-confirmed histological evidence for retatrutide remains unavailable and requires further validation. Retatrutide and tirzepatide are also being evaluated in high-risk MASLD outcome studies such as SYNERGY-OUTCOMES, which focuses on major adverse liver outcomes [46]. Given that the existing evidence for retatrutide is still mainly derived from liver fat reduction and imaging endpoints, it is better placed for discussion in the context of future therapeutic directions and combination/sequential therapy prospects, rather than as a core drug for mature clinical recommendation at this stage. Key clinical evidence and regulatory/development status, along with evidence maturity, are summarised in Table 2.

## 7. Integration of Key Clinical Evidence and Patient Stratification

### 7.1. From Randomised Controlled Trials to Clinical Decision-Making

From the perspective of available randomised controlled studies, the evaluation of MASH drugs is no longer focused solely on changes in body weight, liver fat, or transaminases; histological endpoints and subsequent clinical translational value are becoming more important determinants. Resmetirom and semaglutide have reached FDA accelerated approval, with their histological evidence mainly derived from the phase 3 MAESTRO-NASH and ESSENCE studies [11,12,13,14], representing therapeutic directions with a relatively high degree of evidence maturity and clinical translation. Although tirzepatide has not yet received approval for the MASH indication, the phase 2 SYNERGY-NASH study has demonstrated notably favourable histological improvement results, suggesting that incretin-based drugs have considerable therapeutic and developmental potential in obesity- or insulin resistance-dominant MASH [18]. Drugs such as survodutide, pegozafermin, and efruxifermin represent the multi-axis integrative therapy and FGF21 analogue directions, respectively, with positive phase 2 or 2b histological evidence and are in the stage of advancing to subsequent pivotal studies [15,16,17]. A 2025 systematic review and network meta-analysis further indicates that the relative performance of different drugs on the two histological endpoints of fibrosis improvement and MASH resolution is not entirely consistent, suggesting that clinical evaluation should not be based solely on a single endpoint or trial phase [67]. Therefore, when evaluating MASH drugs, the strength of histological endpoints, regulatory approval status, the state of ongoing confirmatory studies, and phenotypic suitability for patients should also be considered together.

### 7.2. Phenotype-Guided Drug Selection

From the perspective of stratified treatment, abnormalities in glucose metabolism and other cardiometabolic risk factors are closely associated with fibrosis severity and adverse clinical outcomes; patient stratification and treatment selection should therefore be based first on metabolic phenotype identification [25,68,69]. For patients with marked obesity or overweight accompanied by type 2 diabetes or significant insulin resistance, incretin-based drugs can be considered a metabolic intervention direction of interest. When greater weight reduction and a higher likelihood of histological improvement are prioritised, tirzepatide has considerable therapeutic and developmental potential, supported by its prominent phase 2 histological evidence [18]; however, it is still better discussed as a key investigational drug at this stage. If emphasis is placed on approved indications and clinical accessibility, semaglutide has more robust phase 3 histological evidence and regulatory approval status [13].

For patients with non-cirrhotic F2–F3 fibrosis and concomitant elevations in LDL-C or triglycerides, the clinical positioning of resmetirom is relatively well defined, with its phase 3 histological evidence derived from the MAESTRO-NASH study [11]; the AASLD practice update and expert recommendations also emphasise that patient selection should integrate fibrosis stage, lipid profile, drug interactions, and safety monitoring [36,37]. For patients with a high hepatic fat burden or high risk of fibrosis progression, FGF21 analogues can be considered a direction of interest; pegozafermin and efruxifermin have demonstrated positive histological signals [15,17], and relevant phase 3 programmes are ongoing.

For cost-sensitive patients with marked insulin resistance who can accept risks such as weight gain, pioglitazone remains a stratified alternative [33,34]. For those with type 2 diabetes-dominant disease who are more concerned with comprehensive cardiorenal metabolic benefits, SGLT2 inhibitors have certain clinical utility [31,32]. Therefore, drugs targeting DNL and triglyceride synthesis that are still in the development stage, as well as some multi-receptor agonists, are currently better positioned as important exploratory directions for future combination therapy, sequential therapy, and precision-stratified intervention, rather than as mature first-line treatment options. To make the phenotype-based treatment logic clearer, the drug selection framework is presented in Table 3 as a decision-oriented sequence from dominant clinical phenotype to therapeutic goal, candidate drug class, alternatives or combinations, and key safety considerations.

### 7.3. Safety and Clinical Considerations

Beyond metabolic phenotype and fibrosis stage, safety, tolerability, and long-term adherence are also important considerations for stratified selection of MASH drugs. The main adverse effects of GLP-1 receptor agonists, GIP/GLP-1 dual receptor agonists, and GLP-1/GCGR dual receptor agonists are gastrointestinal in nature, including nausea, vomiting, diarrhoea, constipation, and decreased appetite; some of these are related to dose escalation and the early phase of treatment [13,61,70]. Large-scale weight loss, cardiovascular outcome studies, and MASH-related trials all suggest that while incretin-based drugs provide benefits in weight reduction and metabolic parameters, attention should also be paid to gastrointestinal tolerability, discontinuation rates, long-term adherence, and the risk of gallbladder or biliary tract disease [16,71,72].

SGLT2 inhibitors have high clinical utility in patients with type 2 diabetes and high cardiorenal metabolic risk [73,74,75], but attention should also be paid to risks such as genitourinary tract infections, volume depletion, decreases in blood pressure, an initial decline in estimated glomerular filtration rate, and rare cases of ketoacidosis [76]. In elderly patients, or those with inadequate food intake, dehydration, perioperative status, or severe infections, treatment should be temporarily withheld or adjusted accordingly. Thus, in patients with MASH and type 2 diabetes, the hepatic benefits of SGLT2 inhibitors should be weighed together with their cardiorenal protective effects and potential adverse reactions in a comprehensive judgement [31].

Pioglitazone has an evidence base for improving insulin sensitivity and NASH/MASH histological activity, but its clinical use is limited by weight gain, fluid retention, oedema, heart failure risk, and fracture risk. It is therefore better suited as a stratified alternative for patients with marked insulin resistance who are cost-sensitive and have no overt risk of heart failure or high fracture risk, rather than as a universal option for all MASH patients [33,34]. Resmetirom, as a liver-targeted THR-β agonist, requires attention to common adverse effects such as diarrhoea and nausea, as well as monitoring for drug interactions, changes in lipid profile, liver function, and thyroid-related parameters [11,36,37]. Individualised assessment is particularly important in patients receiving concomitant statin therapy, with pre-existing thyroid disease, or on multiple medications.

FGF21 analogues and multi-receptor agonists have shown certain clinical signals for MASH resolution, liver fat reduction, or fibrosis improvement, but longer-term safety and real-world tolerability still require more data [16,17,29]. Current studies suggest that common adverse effects of these drugs mainly include gastrointestinal reactions, injection site reactions, or changes in metabolic parameters. As treatment duration extends and combination therapy scenarios increase, their long-term adherence and potential effects on cardiometabolic parameters continue to require observation [15,77]. Therefore, the choice of metabolism-directed therapy for MASH should not be based solely on a single histological efficacy endpoint or the magnitude of weight reduction, but rather on a comprehensive judgement integrating the patient’s metabolic phenotype, fibrosis stage, comorbidity status, drug accessibility, safety, tolerability, and long-term adherence.

## 8. Limitations and Future Directions

Several issues in current pharmacological research on MASLD/MASH still need to be addressed, including endpoint selection, patient screening, population coverage, and real-world application. Regarding endpoints, most approved drugs and those in key stages of development are based primarily on histological surrogate endpoints rather than directly on clinical outcome endpoints such as hepatic decompensation, liver transplantation or liver-related mortality. At the same time, there remains some inter-reader variability in the assessment of histological features such as hepatocyte ballooning in MASH diagnosis [78]. Previous prospective outcome studies and natural history studies have shown that fibrosis stage is closely associated with long-term outcomes such as hepatic decompensation and death [79,80]. This also implies that whether subsequent confirmatory studies can demonstrate that histological improvement translates into long-term clinical benefit will directly affect the clinical translational value of the relevant drugs.

Regarding patient selection, not all patients in the real world are suitable for or willing to undergo liver biopsy. How to more accurately identify patients likely to benefit from treatment using non-invasive markers is a key issue in clinical implementation [2,26,28].

Regarding the coverage of clinical study populations, most current studies are focused mainly on non-cirrhotic F2–F3 fibrosis populations, while evidence for patients with compensated or decompensated cirrhosis, complex metabolic comorbidities, elderly patients, and long-term combination therapy strategies remains relatively insufficient [49,81,82].

Another important limitation is that the first axis of this framework is most directly applicable to patients in whom obesity, visceral adiposity, type 2 diabetes, or marked insulin resistance represents a dominant therapeutic driver. It should not be interpreted as a universal sequence in which weight loss necessarily precedes or dominates the other therapeutic axes. In normal-weight or “lean” MASLD/MASH, excessive weight reduction may be inappropriate, and management may need to focus instead on cardiometabolic risk factors, visceral adiposity, diet quality, physical activity, fibrosis risk, intrahepatic lipid handling, or inflammatory-fibrotic activity [25,26,27]. Therefore, the three-axis framework should be applied as a phenotype-sensitive model rather than as a weight-centred algorithm for all patients.

Beyond evidence itself, drug accessibility and economic burden also affect real-world implementation of long-term MASLD/MASH management. Although GLP-1 receptor agonists and some novel multi-receptor agonists have demonstrated strong potential for metabolic and histological benefit, their cost, requirement for long-term treatment, and adherence issues may limit their application in broader MASLD populations. Future research, in addition to focusing on histological and clinical outcomes, will also need to further assess the cost-effectiveness and real-world accessibility of different treatment strategies [83,84].

Future MASLD/MASH therapy may gradually shift from efficacy validation of single-target drugs towards combination or sequential treatment strategies involving multi-receptor agonists, liver-targeted drugs, and diabetes medications. In patients with prominent insulin resistance or concomitant type 2 diabetes, the combination or sequential use of pioglitazone with GLP-1 receptor agonists or SGLT2 inhibitors remains a direction for further research [85]. At the same time, pharmacotherapy should still be built upon a foundation of lifestyle management; lifestyle factors such as the Mediterranean diet, coffee consumption, and reduction in unhealthy dietary burden can serve as important components of comprehensive metabolic risk intervention [86,87,88]. If future studies can further integrate body weight, glucose and lipid metabolic parameters, non-invasive fibrosis assessment and histological endpoints, and establish reproducible risk stratification models, this will help shift the selection of MASLD/MASH drugs from empirical judgement towards phenotype-based stratified intervention.

## 9. Conclusions

The core focus of pharmacotherapy for MASLD/MASH has gradually shifted from simply reducing liver fat or improving transaminases towards a comprehensive assessment of histological endpoints, fibrosis risk, and metabolic phenotype. Within this spectrum, MASH represents an active and progressive form of MASLD, characterised by hepatocellular injury, inflammation, and an increased risk of fibrosis progression. Therefore, future treatment strategies should not simply rank drugs by class or a single efficacy endpoint, but rather pay greater attention to the correspondence between disease mechanisms, fibrosis stage, cardiometabolic risk, and patient subtypes [23,24,25].

Focusing on the weight–insulin resistance–substrate load axis, the intrahepatic lipid reprogramming axis, and the inflammation–fibrosis transition and multi-axis integration axis, this review has discussed the mechanistic positioning, evidence maturity, and potential target populations of different drugs. For patients with obesity, type 2 diabetes, or predominant insulin resistance, upstream metabolic unloading may represent an important therapeutic foundation, whereas in normal-weight or lean MASH the role of weight-centred treatment should be individualised rather than assumed; for those with non-cirrhotic F2–F3 fibrosis and concomitant dyslipidaemia, intrahepatic lipid reprogramming therapy has clearer translational value; for patients with higher MASH activity, greater risk of fibrosis progression or insufficient response to metabolic improvement alone, multi-axis integrative therapy may offer a novel disease-modifying approach. A 2024 data-driven phenotyping study further suggests that patients with MASLD can present distinct liver-specific and cardiometabolic profiles, supporting a future shift from empirical single-agent selection towards precise, phenotype-based stratified intervention [27,69].

In summary, pharmacotherapy for MASLD/MASH has entered a new phase that equally emphasises metabolic targeting, histological endpoints, and patient stratification. Subsequent research is still needed to further clarify the relationship between histological surrogate endpoints and long-term liver-related outcomes, and to establish more reliable and reproducible non-invasive pathways for risk stratification and efficacy monitoring. With the advancement of key phase 3 studies, real-world evidence, and combination or sequential treatment strategies, MASLD/MASH therapy is expected to gradually evolve towards an individualised management model based on pathological stage, metabolic phenotype, and treatment goals [26,28,78].

## Figures and Tables

**Table 1:** Pathogenesis-informed three-axis framework for phenotype-guided therapy of MASH within the MASLD spectrum.

Dominant Pathological Axis	Core Pathological Nodes	Representative Drugs and Supporting References	More Relevant Clinical Phenotype	Evidence Stage
Weight-insulin resistance-substrate load axis	Obesity, visceral adiposity, insulin resistance, increased hepatic FFA/glucose influx	semaglutide * [13,14,29]; tirzepatide [18,30]; SGLT2 inhibitors [31,32]; pioglitazone [33,34,35]	Patients with obesity/overweight, T2D, or metabolic syndrome; evidence mainly derives from F2–F3 MASH, with high-risk F1 requiring individualised discussion	A–B range
Intrahepatic lipid reprogramming axis	DNL activation, triglyceride synthesis/storage, lipotoxicity, abnormal lipoprotein export, mitochondrial dysfunction	resmetirom * [11,12,36,37]; pegozafermin [17,38]; efruxifermin [15,39,40,41]; denifanstat [42]; ervogastat ± clesacostat [43]	Non-cirrhotic F2–F3 MASH with dyslipidaemia, high liver-fat burden, or progression risk	A–B range
Inflammation-fibrosis transition and multi-axis integration axis	MASH activity, inflammatory amplification, hepatic stellate-cell activation, ECM deposition	survodutide [16,44]; efinopegdutide [45]; retatrutide [29,46]; FGF21 analogues with cross-axis effects [15,17,39,47,48]	Higher MASH activity, F3/high progression risk, or need for broader metabolic and hepatic effects	B–C range

Notes. Evidence maturity: A, regulatory approval and/or positive phase 3 histological evidence; B, phase 2/2b biopsy-based histological evidence requiring pivotal validation; C, imaging-based, biomarker-based or exploratory evidence without mature histological validation. The clinical phenotypes listed in the table should be interpreted using routine metabolic, lipid, and fibrosis assessments, including body habitus, visceral adiposity, glucose metabolism, lipid profile, liver fat burden, histological activity, and non-invasive fibrosis evaluation. * indicates FDA accelerated approval for MASH within this manuscript framework; local labels may differ. This table is intended for conceptual and translational positioning and is not a formal treatment guideline. FFA, free fatty acid; DNL, de novo lipogenesis; ECM, extracellular matrix.

**Table 2:** Key clinical evidence and regulatory/development status of metabolism-directed drugs for MASH.

Drug	Key Published Evidence and References	Main Findings	Reported Regulatory/Development Status
resmetirom *	MAESTRO-NASH; mainly F2–F3; 52 weeks [11,12,36,37]	MASH resolution and fibrosis improvement were superior to placebo.	FDA accelerated approval; confirmatory outcomes follow-up ongoing.
semaglutide 2.4 mg *	ESSENCE; F2–F3 MASH; 72-week interim analysis [13,14,57]	Both co-primary histological endpoints were superior to placebo.	FDA accelerated approval; long-term ESSENCE follow-up ongoing.
tirzepatide	SYNERGY-NASH; phase 2; F2–F3 MASH; 52 weeks [18,30]	MASH resolution and fibrosis improvement were higher than placebo.	Not approved for MASH; pivotal/outcomes studies ongoing or planned.
survodutide	Phase 2 study; MASH with fibrosis; 48 weeks [16,44]	Higher MASH improvement without fibrosis worsening; positive fibrosis signal.	Phase 3 LIVERAGE programme ongoing.
dapagliflozin	Biopsy-guided RCT; MASH; 48 weeks [31]	MASH improvement, resolution, and fibrosis improvement favoured dapagliflozin.	No MASH-specific approval; relevant in T2D/cardiorenal comorbidity.
pegozafermin	ENLIVEN phase 2b; MASH with fibrosis [17,38]	Improved fibrosis, MASH resolution, liver fat, and metabolic markers.	Phase 3 ENLIGHTEN programmes ongoing/initiated.
efruxifermin	HARMONY phase 2b and longer follow-up [15,39,40,41]	Sustained MASH resolution and fibrosis-improvement signals.	Phase 3 SYNCHRONY programmes ongoing/initiated.
denifanstat	FASCINATE-2; phase 2b; F2–F3 MASH [42]	Improved disease activity and MASH-resolution-related histology.	Positive phase 2b evidence; FDA Breakthrough Therapy designation; further development under evaluation.
ervogastat ± clesacostat	MIRNA; phase 2 [43]	Early liver-fat and histological signals; ACC-related triglyceride elevation requires monitoring.	Further validation required; mainly combination/pathway-specific positioning.
retatrutide	Phase 2 obesity/MASLD imaging substudy [29,46]	Marked MRI-based liver-fat reduction; high-dose groups achieved steatosis resolution in most participants.	Biopsy-confirmed MASH histological endpoints remain unavailable and require validation; outcome studies are ongoing.

Notes. This table summarises key published clinical evidence and publicly reported regulatory/development status. Regulatory and development status may vary across regions and should be interpreted according to local labels and updated guidelines. * indicates drugs with FDA accelerated approval for MASH in this manuscript framework; local regulatory labels may differ.

**Table 3:** Decision-oriented phenotype-guided pharmacotherapeutic framework for MASH in the MASLD spectrum.

Step 1: Dominant Clinical Phenotype	Step 2: Core Therapeutic Goal	Step 3: Main Drug Classes to Consider	Step 4: Potential Alternatives or Combinations	Step 5: Key Safety or Implementation Issues
Obesity or marked overweight with insulin resistance	Substantial weight loss and upstream metabolic unloading	semaglutide 2.4 mg *; tirzepatide (developmental, strong phase 2 histology)	SGLT2 inhibitors; lifestyle intervention; pioglitazone in selected patients	GI tolerability, discontinuation risk, and gallbladder/biliary events.
Non-cirrhotic F2–F3 MASH with LDL-C or triglyceride elevation	Improve liver fat, lipid profile, and histological endpoints	THR-β agonist resmetirom *	FGF21 analogues; lifestyle and standard lipid-management therapy	Drug interactions; liver function, lipid profile, and thyroid-related monitoring.
More severe hepatic phenotype or F3/high progression risk	Maximise MASH resolution and fibrosis improvement signals	FGF21 analogues such as pegozafermin and efruxifermin	Survodutide and other multi-axis/multi-agonist approaches	Phase 3 evidence, long-term safety, and real-world tolerability remain under evaluation.
T2D with elevated cardiovascular or renal risk	Improve glycaemia and cardiorenal risk while reducing metabolic substrate load	SGLT2 inhibitors; GLP-1RAs	tirzepatide (developmental for MASH); pioglitazone in carefully selected patients	Genitourinary infection, volume depletion, initial eGFR dip, and rare ketoacidosis with SGLT2i.
Marked insulin resistance with GLP-1RA intolerance or inaccessibility	Improve insulin sensitivity and MASH activity	pioglitazone	SGLT2 inhibitors; intensive lifestyle intervention	Use cautiously or avoid in patients with heart failure, oedema, or high fracture risk; monitor for weight gain and fluid retention.

Notes. The rows are not mutually exclusive; real-world patients may meet several phenotypic patterns simultaneously. The table is intended to function as a practical decision-oriented summary rather than as a rigid flowchart or prescribing algorithm. * indicates drugs with FDA accelerated approval for MASH at the time of manuscript preparation within this manuscript framework; local regulatory labels may differ. Other drugs may be investigational for MASH or used in selected comorbidity contexts. This table is intended for conceptual positioning and is not a prescribing guideline.

## Data Availability

No new data were created or analysed in this study. Data sharing is not applicable to this article.
